# Comparative Transcriptomics of Malaria Mosquito Testes: Function, Evolution, and Linkage

**DOI:** 10.1534/g3.117.040089

**Published:** 2017-02-02

**Authors:** Bryan J. Cassone, Raissa G. G. Kay, Matthew P. Daugherty, Bradley J. White

**Affiliations:** * Department of Biology, Brandon University, Manitoba R7A 6A9, Canada; † Department of Entomology, University of California, Riverside, California 92521; ‡ Graduate Program in Genetics, Genomics, and Bioinformatics, University of California, Riverside, California 92521

**Keywords:** RNA sequencing, demasculinization, *Anopheles*, X-linked

## Abstract

Testes-biased genes evolve rapidly and are important in the establishment, solidification, and maintenance of reproductive isolation between incipient species. The *Anopheles gambiae* complex, a group of at least eight isomorphic mosquito species endemic to Sub-Saharan Africa, is an excellent system to explore the evolution of testes-biased genes. Within this group, the testes are an important tissue in the diversification process because hybridization between species results in sterile hybrid males, but fully fertile females. We conducted RNA sequencing of *A. gambiae* and *A. merus* carcass and testes to explore tissue- and species-specific patterns of gene expression. Our data provides support for transcriptional repression of X-linked genes in the male germline, which likely drives demasculinization of the X chromosome. Testes-biased genes predominately function in cellular differentiation and show a number of interesting patterns indicative of their rapid evolution, including elevated *dN/dS* values, low evolutionary conservation, poor annotation in existing reference genomes, and a high likelihood of differential expression between species.

Due to male–male, male–female, and intragenomic conflict, testes-biased genes (*i.e.*, expressed exclusively in or predominately in male testes relative to other tissues) exhibit a number of extraordinary evolutionary properties (Meiklejohn *et al.* 2003; Ellegren and Parsch 2007; Mank *et al.* 2014). First, testes genes are often subject to strong positive selection, leading to faster sequence evolution relative to genes with alternative expression profiles (Oliver *et al.* 2010; Parsch and Ellegren 2013). Second, the testes are the birthplace of a disproportionate number of novel genes (Kaessmann 2010; Zhao *et al.* 2014). Indeed, turnover rate for testes-biased genes and gene families is highly accelerated relative to other classes of genes (Zhang *et al.* 2007; Assis *et al.* 2012). Finally, in species where the male is heterogametic, genes upregulated in the testes are often unequally distributed across the genome, with a paucity located on the X chromosome relative to the autosomes (Reinke *et al.* 2000; Parisi *et al.* 2003; Storchova and Divina 2006; Sturgill *et al.* 2007; Prince *et al.* 2010; Magnusson *et al.* 2012). Demasculinization of the X is a pattern found across diverse taxa and is likely driven by a lack of dosage compensation, meiotic sex chromosome inactivation (MSCI), or a combination of the two (Campbell *et al.* 2013).

Due, in part, to their rapid evolution and uneven genomic distribution, testes-biased genes are often critical to the establishment, solidification, and maintenance of reproductive isolation between incipient species. Moreover, Haldane’s rule states that if only one sex of hybrids is sterile or inviable it will be the heterogametic sex (Haldane 1922); diverse taxa including birds (Gray 1958), *Drosophila* (Bock 1984), *Lepidoptera* (Presgraves 2002), mammals (Craft 1938; Gray 1972), and plants (Brothers and Delph 2010) with both XY and ZW sex determination systems largely obey the rule (Coyne and Orr 2004). Indeed, testes defects that result in reduced hybrid male fertility normally manifest before other intrinsic reproductive isolating barriers evolve (Orr 1997; Brothers and Delph 2010; Schilthuizen *et al.* 2011). Hybrid male sterility is generally characterized by failed or reduced sperm development (Howard *et al.* 2009) and/or the reduced growth of reproductive tissues, resulting in smaller testes and lower sperm production (Møller 1989; Hardy *et al.* 2011; Turner *et al.* 2012).

Many of the unique properties ascribed to testes-specific genes have been generalized based on data from select model organisms. With the constantly lowering cost of generating high-throughput sequence data, it is now feasible to determine if these insights are consistent across taxa. The *Anopheles gambiae* complex, a group of at least eight morphologically indistinguishable mosquito species endemic to Sub-Saharan Africa for which extensive genomic resources are available, is a good system to explore the evolution of testes-biased genes. Indeed, nearly all parental crosses among the sibling species result in sterile hybrid males, but fully fertile females (Davidson 1969, Davidson *et al.* 1970). Here, we combine tissue-specific RNA sequencing from two *A. gambiae* complex species (*A. coluzzii* and *A. merus*) with recently published whole genome sequences of three other *Anopheles* species (Neafsey *et al.* 2015) to investigate the evolution of testes genes over multiple phylogenetic timescales. Enabled by a chromosome-based genome assembly, we also analyzed the genomic distribution and expression profiles of testes genes. In addition to broad evolutionary insights, studying testes-biased genes in *Anopheles* has public health relevance because anophelines are the exclusive vectors of mammalian malaria. Indeed, the two focal species of this study are major vectors of the malignant malaria species, *Plasmodium falciparum*, across their ranges in Sub-Saharan Africa (White *et al.* 2011). Genes critical to *Anopheles* sperm and testes development may serve as targets for mosquito population suppression via genetically-based sterile insect techniques (Alphey *et al.* 2010). Despite their importance, no anopheline testes transcriptomes have been fully characterized, slowing identification of candidate gene targets.

Our analysis of the *A. coluzzii* and *A. merus* testes and carcass transcriptomes revealed a number of interesting patterns, including demasculinization of the X chromosome, poor conservation of tissue-specific (including testes) genes, rapid evolution of testes-biased genes, and a startlingly high number of putative testes transcripts that are unannotated and/or completely missing from the reference genome assembly. Additionally, we positively identified hundreds of testes-specific genes, providing a long list of candidate male fertility genes for functional testing.

## Materials and Methods

### Mosquito maintenance

*A. coluzzii* (from the Mali-NIH colony) and *A. merus* (from the MAF colony) mosquitoes were maintained under controlled conditions of 27°, 65% relative humidity, and a 12-hr light/dark cycle with 1 hr dawn and dusk transitions. After hatching, 200 larvae were counted and placed into a plastic tray containing 1 L ddH20. Each tray was fed ∼200 mg of a 4:1 mixture of finely ground fish pellets to baker’s yeast daily. Adult males were kept isolated from females until dissection.

### Experimental design

A total of 200 *A. coluzzii* or *A. merus* 5–10-d-old virgin males were randomly selected from the Mali-NIH and MAF colonies, respectively. The male reproductive tract was dissected from each mosquito and the testes were isolated under 400× magnification on a compound microscope and placed in TRIzol (the accessory glands were not included with the testes). The remaining carcass was frozen in TRIzol at −80° until RNA extraction. These experiments were replicated four times using mosquitoes from different cohorts for a total of 16 samples: two tissues (testes and carcass) from two species (*A. coluzzii* and *A. merus*), and four replications of each.

### RNA isolation and cDNA library preparation

For each sample, total RNA was extracted from pools of 200 mosquito testes or 20 carcasses, using TRIzol (Life Technologies, Carlsbad, CA). RNA quantity and quality were assessed using the BioAnalyzer 2100 (Agilent Technologies, Santa Clara, CA). RNA (13 ng per sample) was used to generate cDNA libraries for RNA sequencing using the NEBNext Ultra RNA library prep kit for Illumina, following the manufacturer’s protocol (New England BioLabs, Ipswich, MA). Quantification and quality inspection of double-stranded cDNA was carried out using the BioAnalyzer 2100. Samples were diluted to 2 nM and pooled to generate two multiplexed cDNA libraries.

### Illumina sequencing and read preprocessing

The pooled cDNA libraries were sequenced on two flow cell lanes using the Illumina HiSequation 2500 platform at the University of California, Riverside Genomics Core. The mean library insert sequence size was 277 bp and both ends of the library were sequenced to generate 100 bp raw paired-end reads. Raw reads were imported into CLC Genomics Workbench (v6.5.1; CLC bio, Aarhus, Denmark) where adapter indexes and poly(A) tails were trimmed (Ambiguous limit = 2, quality limit = 0.05). Quality assessment of the normalized data file included hierarchical clustering of samples (measure: Euclidean distance; clusters: average linkage) and principal component analyses (PCA) in CLC bio. The raw sequence reads can be retrieved from the NCBI short sequence read archive under the accession number SRP047496.

### Tissue-specific transcriptomic analysis

The *A. coluzzii* (formerly *A. gambiae* M Form) (AgamP4.2; 13,624 genes) and *A. merus* (AmerM1.2; 14,415 genes) reference gene sets were retrieved from Vectorbase (Megy *et al.* 2012). To obtain read counts, preprocessed reads were aligned to the reference gene sets using the map to reference function of the CLC bio Genomics Workbench and the following parameters: similarity fraction = 0.95; length fraction = 0.95; default settings herein. Read counts were normalized by calculating the number of unique (*i.e.*, unambiguous) reads per kilobase of exon model per million mapped reads (RPKM) (Mortazavi *et al.* 2008).

The *A. coluzzii* and *A. merus* datasets were each filtered to contain only the expressed gene subset, defined as having a minimum RPKM value of four for all replicates in at least one tissue. Genes differentially expressed between the testes and carcass tissues were identified using DESeq2 (Anders and Huber 2010), which determines changes in transcript accumulation based on a negative binomial distribution model. Significance was defined at an FDR < 0.05 (Benjamini and Hochberg 1995) for this and all other analyses, unless otherwise specified. Differentially expressed genes were categorized as tissue-specific if they had RPKM values less than one for all replicates in the under expressed tissue, thereby ensuring that the tissue-specific designation is robust and not influenced by variability in total read counts across samples. The overrepresentation test in the PANTHER (protein annotation through evolutionary relationship) Classification System (Mi *et al.* 2013) was used to identify enriched *A. coluzzii* biological processes. This large-scale gene function analysis is based on the *A. gambiae* Gene Ontology (GO) database released December 28, 2016. The significance threshold was set at *P* < 0.05 with Bonferroni correction for multiple testing.

### Species-specific transcriptomic analysis

To examine gene expression differences between *A. coluzzii* and *A. merus* testes and carcass tissues, Bayes-moderated *t* tests (FDR < 0.05) were carried out on each tissue independently using the limma package in R (Ritchie *et al.* 2015). Given the interspecies comparison, differential expression was based on RPKM values and determined for the subset of 9719 *A. coluzzii* genes that had a predicted *A. merus* ortholog. For each contrast, only the expressed genes (refer to Tissue-specific transcriptomic analysis section) in at least one species were represented in the final dataset.

### dN/dS analysis of the testes

To compare the evolutionary rates of testes- and carcass-biased genes, we retrieved the ratios of nonsynonymous-to-synonymous mutations (*dN/dS*) between *A. coluzzii* and three other anophelines (*A. merus*, *A. christyi*, and *A. epiroticus*) from VectorBase (Giraldo-Calderón *et al.* 2015). The *dN/dS* ratios of the one-to-one orthologs were compared among each of three expression profiles (expressed, enriched, and specific) and between tissues (carcass and testes) using three-way ANOVA (Crawley 2007), and *dN/dS* values were fourth-root transformed to meet test assumptions. We also determined the proportion of *A. coluzzii* genes that retained a one-to-one ortholog in two anophelines (*A. merus* and *A. melas*) and three more evolutionary distant dipterans (*Drosophila melanogaster*, *Aedes aegypti*, and *Culex quinquefasciatus*).

### Identification of potential unannotated and Y-linked genes

To identify unannotated testes genes and potential Y-linked genes in *A. coluzzii*, we performed two complementary analyses. First, we assembled the *A. coluzzii* testes transcriptome *de novo* and searched for contigs (*i.e.*, partial or complete mRNA sequences) not present in the current *A. coluzzii* genome assembly. The Trans-AByss pipeline was utilized since it robustly assembles even contigs with low expression levels (Robertson *et al.* ‎2010). k-mer assemblies were generated using AByss v1.3.2 with minimum mean k-mer coverage of a unitig of two (Birol *et al.* 2009). Individual assemblies were performed using all even k-mer lengths between 52 and 96 (23 assemblies total). Trans-AByss v1.4.4 was used merge the individual assemblies into a meta-assembly (Robertson *et al.* 2010). Redundant sequences were removed from the merged multiple k-mer assembly using CAP3 (Huang and Madan 1999). Each assembled contig was then mapped against the current genome build (AcolM1; 22.46 Mb) using BLASTn (*E*-value < 1 × 10^−6^). Contigs were grouped based on whether they (1) mapped to regions of the genome annotated as intergenic, (2) mapped to scaffolds that were unplaced, or (3) did not map to the genome at all. OrfPredictor was then used to identify contigs within the above groups that had open reading frames >50% of their length (Min *et al.* 2005). A subset of contigs that mapped to either unplaced scaffolds or did not map to the genome at all were tested for Y-linkage via PCR amplification of male and female DNA from the *A. coluzzii* Mali-NIH colony. To check amplification, PCR products were visualized on a 1.5% agarose gel stained with ethidium bromide. PCR products that amplified in only male DNA were classified as Y-linked.

Second, we generated a *de novo* transcriptome assembly using only testes reads that failed to map to the *A. coluzzii* reference genome. Reads were mapped to the most recent AcolM1 genome build, using the map to reference function in CLC bio Genomics Workbench and the following parameters: similarity fraction = 0.90; length fraction = 0.70; default settings herein. Next, the unmapped reads were assembled *de novo* into contigs (≥250 bp) using two independent approaches: (1) the CLC bio algorithm based on de Bruijn graphs and the optimized parameters, defined as word size = 64, bubble size = 500, length fraction = 0.98, and similarity fraction = 0.98; and (2) Oases v0.2.08 with k-mer sizes of 53, 59, 65, 71, 77, 83, and 89 (Schulz *et al.* 2012). To obtain the set of nonredundant transcripts for each assembly, transcripts with ≥90% sequence similarity were collapsed into clusters and the longest read retrieved using CD-HIT-EST (Li and Godzik 2006). The two independent assemblies were collapsed into a final assembly using Minimus2 (Sommer *et al.* 2007). Contigs of microbial origin were identified and removed using BLASTn against the bacteria nonredundant database (*E*-value < 1 × 10^−10^) and GC content >45%. Testes and carcass reads were then remapped onto the remaining contigs and tested to assess differential expression among tissues. As described above, a subset of contigs were tested for Y-linkage via PCR amplification.

### Data availability

The raw sequence reads are deposited in the NCBI short sequence read archive under the accession number SRP047496.

## Results and Discussion

We performed RNA sequencing separately on the testes and carcass from two species within the *A. gambiae* complex of mosquitoes: *A. coluzzii* (formerly *A. gambiae* M Form) and *A. merus*. Two cDNA libraries consisting of 16 pooled samples were sequenced, which generated 475 million paired-end reads of 100 bp. After demultiplexing and trimming poor quality reads, adapters, and poly(A) sequences, 75% of reads were retained (between 13.3 and 30.8 million nonredundant reads per sample). Preprocessed reads were mapped to *A. coluzzii* and *A. merus* gene sets and RPKM normalized transcript expression levels were calculated for each sample independently. Data from one *A. coluzzii* testes and one *A. coluzzii* carcass replicate were omitted prior to subsequent analysis due to low read mapping counts and poor average quality indices.

Two-dimensional PCA were generated to visualize the relationships among the *A. coluzzii* and *A. merus* cDNA libraries derived from testes and carcass tissues (Supplemental Material, Figure S1). The libraries for each tissue clustered near one another and discretely from the other tissue. This spatial distribution confirms that the global expression profiles of testes and carcass tissues vary dramatically from one another.

### Demasculinization of the X

#### Paucity of testes-biased genes on X:

For each species, we placed genes into six categories based on their expression patterns: (1) testes expressed (TX, expressed in the testes); (2) testes enriched (TN, significantly higher abundance in testes relative to carcass); (3) testes specific (TS, expressed only in the testes); (4) carcass expressed (CX); (5) carcass enriched (CN); and (6) carcass specific (CS). These categories were not mutually exclusive; therefore, some genes were grouped into more than one category. The gene counts for each category, broken down by species and chromosomal arm, are listed in Table 1. In both species, chi-squared tests (χ^2^) revealed that the number of TX, TN, and TS genes was underrepresented on the X chromosome (all comparisons: *P* < 0.0001 after Yates correction). In contrast, CX, CS, and CN genes were evenly distributed across the *Anopheles* chromosome arms.

**Table 1:** Chromosomal distribution of genes with the six designated expression profiles in *A. coluzzii* and *A. merus*

Species	Chromosome	TX*^a^*	TN*^b^*	TS*^c^*	CX	CN	CS
*A. coluzzii*	2L	1436	603	100	1298	403	67
	2R	1977	867	122	1779	589	89
	3L	1063	436	85	956	315	54
	3R	1340	589	112	1207	421	63
	X	242	21	17	461	326	41
	UNK	140	51	9	139	41	11
	Total	6198	2567	445	5840	2095	325
*A. merus*	2L	1256	648	84	1211	486	74
	2R	1705	919	103	1572	624	80
	3L	818	453	59	745	281	40
	3R	1151	582	96	1080	433	63
	X	191	26	9	419	302	58
	UNK	31	15	3	27	12	3
	N/A	990	395	173	1020	553	113
	Total	6142	3038	527	6074	2691	431

a Expressed genes had a minimum RPKM value of four for all replicates in at least one tissue.

b DESeq2 was used to identify differentially expressed genes between tissues for each species.

c Differentially expressed genes were defined as tissue-specific if they had RPKM values <1 for all replicates in the underexpressed tissue.

#### X chromosome genes are downregulated in the testes:

**Figure 1:**
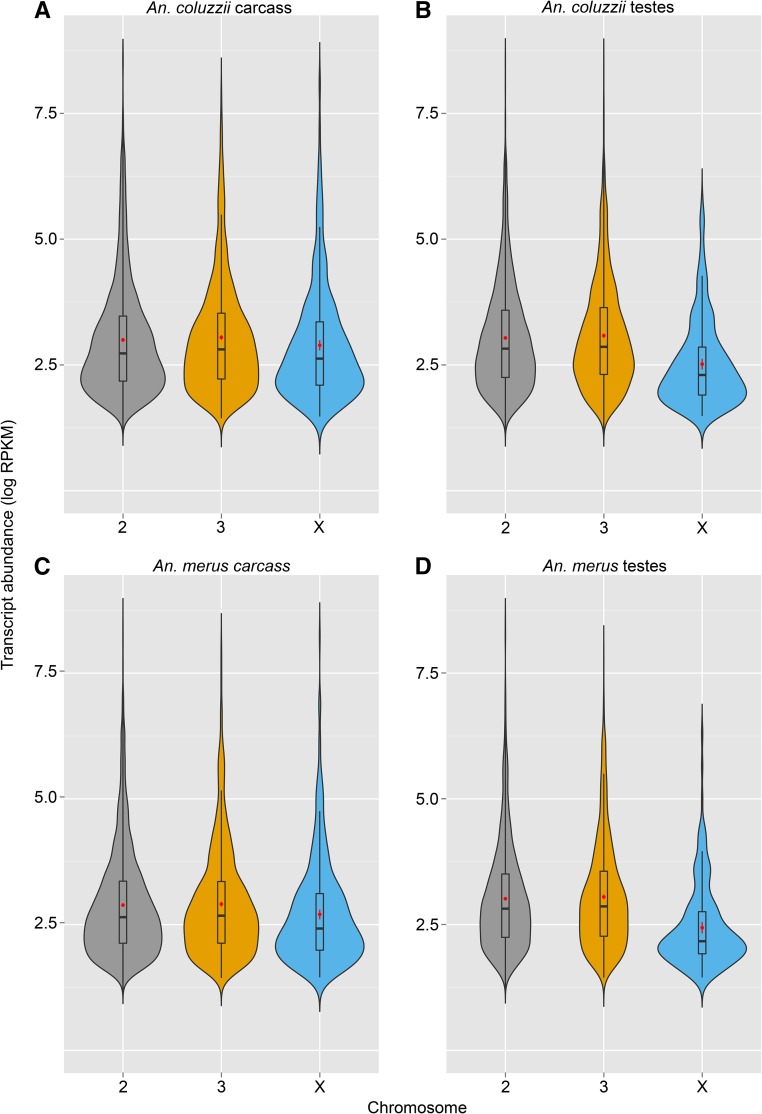
Transcription of the X chromosome is repressed in *Anopheles* testes (A–D). Violin plots show the density (represented by width) of transcript abundances (log RPKM) of all genes located on the 2, 3, and X chromosomes. Within each violin plot is a box plot that shows median, 25th, and 75th percentile values of transcript abundance (±SD). Within each box plot, a red dot denotes the mean transcript abundance (±SEM). All visualizations support X-specific silencing in the male germline, but not soma, of both species.

To determine if X-linked genes are silenced in the male *Anopheles* germline, we first visualized the log RPKM expression levels of genes located on different chromosomes (Figure 1). Mean expression was then analyzed statistically using a three-way ANOVA, with fixed effects of species (*A. coluzzii* or *A. merus*), tissue (carcass or testes), and chromosome (2, 3, or X). Expression levels differed significantly between species (*F*_1,35_ = 29.17; *P* < 0.0001), tissues (*F*_1,35_ = 137.8; *P* < 0.0001), and among chromosomes (*F*_2,35_ = 198.7; *P* < 0.0001), with an interaction between tissue type and chromosome (*F*_2,35_ = 117.48; *P* < 0.0001). In both *A. coluzzii* and *A. merus* carcass, gene expression did not dramatically differ between chromosomes. In contrast, in testes samples, X-linked genes exhibited 61.4% lower mean expression than autosomal genes in *A. coluzzii* and 62.4% lower expression in *A. merus*.

**Figure 2:**
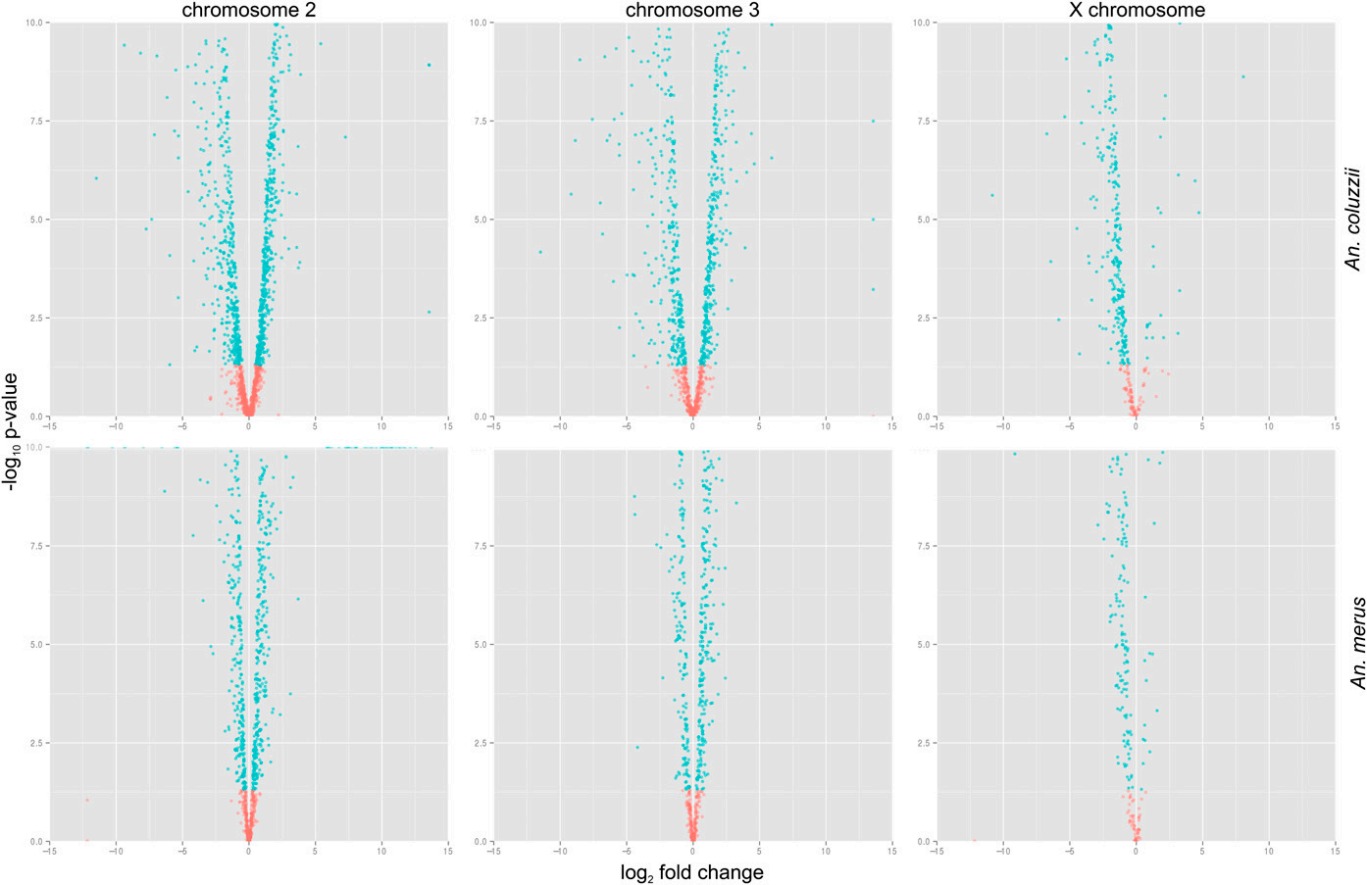
X-linked genes are downregulated in *Anopheles* testes. Volcano plots visualize the fold-difference in individual gene expression between the testes and carcass on each chromosome (2, 3, and X) of *A. coluzzii* and *A. merus*. Each gene is represented by a single dot. The *x*-axis displays log_2_-transformed signal intensity differences between tissues; positive values represent overexpression in testes while negative values represent overexpression in carcass. The *y*-axis displays log_10_-transformed *P*-values associated with ANOVA tests of differential gene expression. Genes are colored in red if they are significantly differentially expressed between the two tissues. In both species, gene expression is balanced between the testes and carcass on the autosomes, while the vast majority of X-linked genes are downregulated in the testes.

We next directly compared the expression of individual genes, sorted by chromosome, in the carcass and testes using volcano plots (Figure 2 and Table S1). In both species, autosomal gene expression is greater in the carcass than in the testes: between 38 and 47% more genes are enriched in the testes. In contrast, on the X the vast majority of genes are significantly downregulated in the testes relative to the carcass (>89%), thus providing additional evidence for X chromosome silencing in the male germline.

#### The cause of X demasculinization:

There are at least two plausible explanations for the observed transcriptional downregulation of the X chromosome in the *Anopheles* male germline. First, it could be due to an absence of dosage compensation: that is, a failure of the heterogametic male (XY) to double the expression of their single X chromosome to match that of the homogametic female (XX). In *Anopheles* somatic tissue, dosage compensation roughly equalizes expression levels of the monosomic X and diploid autosomal genes (Baker and Russell 2011). Recent RNA sequencing to examine *A. gambiae* male-to-female ratios of X expression levels in fourth instar larvae and early pupae suggest incomplete compensation in larvae and no compensation mechanism operating in the male germline of pupae (Rose *et al.* 2016). Our data are largely consistent with this finding; however, two lines of evidence suggest that the low expression of X-linked genes in the testes may not be due to lack of dosage compensation alone. First, absence of dosage compensation should result in ∼50% decrease in X-linked gene expression relative to autosomal gene expression. However, we found that X-linked genes in *Anopheles* testes display >60% lower transcript abundance than autosomal genes, albeit with high variance around this mean. Second, we found a dearth of X-linked testes-specific genes, which would not be predicted if expression levels were simply lowered via a lack of dosage compensation, although it should be noted that we did not have power to detect testes-specific genes with extremely low expression levels (RPKM < 4).

The pattern we observed could be explained by a lack of dosage compensation combined with MSCI, where the male X and Y chromosomes are transcriptionally silenced in primary spermatocytes, and remain repressed for the duration of spermatogenesis (Campbell *et al.* 2013). This phenomenon has been observed in diverse eukaryotes, including mammals (Richler *et al.* 1992), grasshoppers (Cabrero *et al.* 2007), and nematodes (Kelly *et al.* 2002). Indeed, Magnusson *et al.* (2012) showed than when active autosome testes promoters are placed onto the *A. gambiae* X, they largely fail to drive expression. In mice, X repression extends into the postmeiotic period (Campbell *et al.* 2013), whereas in *Drosophila*, repression may begin during mitosis (Meiklejohn *et al.* 2011; Landeen *et al.* 2016). The reason for MSCI remains unclear, although it may have evolved to counter the spread of selfish genetic elements, including both sex ratio distorters and transposable elements (Hamilton 1967; Haig and Grafen 1991; Ellis *et al.* 2005; Kelly and Aramayo 2007), or to limit nonhomologous recombination between heteromorphic sex chromosomes (Daish and Grützner 2010). Interestingly, recombination between the X and Y appears to be relatively common in *A. gambiae* complex males (Hall *et al.* 2016). Since we isolated and sequenced whole testes, we cannot comment on when transcriptional repression may start or stop during sperm development. Combining microscopic laser dissection with single-cell RNA sequencing could potentially shed light on the timing and underlying mechanisms of transcriptional repression in the testes.

### X-linked testes genes are multi-copy

Despite demasculinization of the X chromosome, we did identify 17 X-linked testes-specific genes (Table S2). Interestingly, a number of paralogous groups of TS genes are physically clustered on the X chromosome. For example, ∼2 Mb from the X telomere is a cluster of tandemly arrayed, paralogous TS genes: AGAP000133, AGAP000134, and AGAP000135. Microarray expression data from female tissues (Baker *et al.* 2011) shows that these paralogs are also expressed in ovaries, suggesting they may have important functions in germline cells of both sexes, perhaps explaining their retention on the X. Further functional inference is not possible since no annotation exists for any of these genes and no orthologs are present outside of mosquitoes. Additionally, a cluster of five paralogous TS genes are tandemly arrayed on the X at ∼15 Mb and are not expressed in the ovaries: AGAP000816, AGAP000817, AGAP012997, AGAP013424, and AGAP013173 (note that AGAP000816 and AGAP0013173 only show evidence for expression in the testes, but RPKM values were below our formal cutoff for expressed genes). No functional data for these genes is available and they appear evolutionarily recent, as they are unique to the *A. gambiae* complex of mosquitoes. It is tempting to speculate that these genes are critical for proper testes function, and expansion in gene number occurred as a way of achieving sufficient expression during male gametogenesis. A third cluster of five testes-specific genes are tandemly arrayed at 17 Mb: AGAP012998, AGAP013104, AGAP013235, AGAP0013428, and AGAP0013444. No expression data are available for these genes in ovaries. As with the aforementioned clusters, no functional annotation is present and the genes are unique to the *A. gambiae* complex. In summary, despite the harsh transcriptional environment, we find a number of phylogenetically new testes-specific genes present on the X, often tandemly arrayed in multi-copy families. We hypothesize that tandem duplication is the only way for these genes to achieve sufficient expression on the X, and that movement off of the X is selectively favored but is a rarer event than tandem duplication.

### Functions of testes-biased genes

To determine the cellular and molecular functions of testes genes we conducted GO enrichment analysis on the subset of 3012 testes-biased genes (TN and TS) in *A. coluzzii*. We omitted *A. merus* from the functional analysis because its genome is poorly annotated. The PANTHER overrepresentation test revealed 131 enriched terms (see Table S3). Focusing on the subset of 30 terms with the highest magnitude of enrichment (fold change > 2.5), many of these terms could be neatly clustered into functional groups related to DNA replication and repair (*e.g.*, 0006270, 0032508, 0006310, 0006261, 0006974, 0006260, 0006281, and 0006302), cell cycle (*e.g.*, 0051321, 1903046, 0007126, 0022402, 0007049, and 0007346), and nuclear protein localization (*e.g.*, 0006606, 0044744, 0034504, and 0051170). Overall, the transcripts within these testes-biased clusters all share commonality or interrelatedness of function related to cellular differentiation. Thus, their biased expression is not surprising, as spermatogenesis is a continuum of cellular differentiation (Mecklenburg and Hermann 2016).

### Rapid evolution of testes genes

Rapid evolution in testes genes is a common phenomenon across diverse taxa and is likely due to strong selective pressure from sperm competition (Ramm *et al.* 2014), sexual antagonism (Gallach and Betrán 2011), and intragenomic conflict arising from germline pathogens and selfish genetic elements (Meiklejohn and Tao 2010; Madhani 2013). Indeed, several lines evidence point toward the rapid evolution of *Anopheles* testes-biased genes, as discussed in the following four sections.

#### Tissue-specific genes are less likely to have orthologs:

To search for differences in the evolution rates among testes and somatic genes, we first examined whether *A. coluzzii* testes genes were less likely to have orthologs than carcass genes, factoring in their expression profile (*i.e.*, expressed, enriched, or specific). We searched for orthologs in five species at varying evolutionary distances from *A. coluzzii* (listed from closest to most distantly related): *A. merus*, *A. melas*, *C. quinquefasciatus*, *A. aegypti*, and *D. melanogaster*. Remarkably consistent results among tissues were obtained regardless of which species was used for the analysis (Table 2). The frequency of orthologs was analyzed using a generalized linear model with fixed effects of species, tissue type, and expression profile. Quasibinomial error was assumed to cope with overdispersion of the data (Crawley 2007). The frequency of orthologs differed significantly among comparator species (χ^2^ = 287.307; df = 4; *P* < 0.0001), tissue type (χ^2^ = 4.570; df = 1; *P* = 0.0325), and expression profile (χ^2^ = 64.522; df = 2; *P* < 0.0001), with an interaction between tissue type and expression profile (χ^2^ = 11.452; df = 2; *P* = 0.0033). TS genes were much less likely to have an ortholog than either TN or TX genes. To determine if this pattern was simply a consequence of tissue specificity in general, we examined the ortholog rate of CX, CN, and CS genes. Indeed, while no difference in ortholog rate was found between CN and CX genes, CS genes were much less likely to have an ortholog than both CX and CN genes. Our results suggest that narrowness of expression may be correlated with gene birth/death rate in mosquitoes.

**Table 2:** *A. coluzzii* genes with orthologs (one-to-one) based on the six designated expression profiles

Species	Expression Pattern in *A. coluzzii*, % (*n*)					
	TX (6198)*^a^*	TN (2567)	TS (445)	CX (5840)	CN (2095)	CS (325)
*A. merus*	82.1*^b^* (5087)	84.5 (2157)	73.6 (327)	81.7 (4771)	80.6 (1689)	72.9 (237)
*A. melas*	70.4 (4365)	71.1 (1826)	64.9 (289)	70.2 (4100)	69.3 (1451)	63.4 (206)
*A. aegypti*	68.6 (4254)	70.9 (1821)	46.1 (205)	68.6 (4008)	64.8 (1358)	55.1 (179)
*C. quinquefasciatus*	65.9 (4086)	67.4 (1729)	43.8 (195)	66.5 (3885)	63.1 (1323)	52.3 (170)
*D. melanogaster*	65.6 (4066)	68.2 (1751)	36.2 (161)	63.9 (3729)	53.7 (1125)	37.5 (122)

a Total number of genes in *A. coluzzii* genes identified to have the designated expression pattern.

b Percentage of *A. coluzzii* genes identified to have an ortholog, based on expression pattern and comparator species. For instance, 82.1% of *A. coluzzii* testes-expressed genes have an ortholog match to *A. merus*. The total number of *A. coluzzii* ortholog genes is denoted in parentheses.

#### dN/dS values are greater for testes genes:

**Figure 3:**
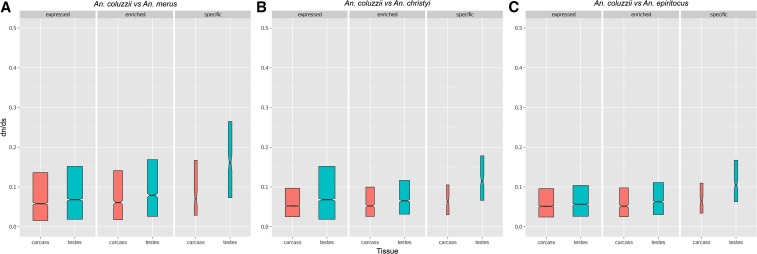
Testes-biased genes show accelerated evolution relative to carcass-biased genes (A–C). Notched box-plots display the median, 25th, and 75th percentile values of *dN/dS* ratios (untransformed) between *A. coluzzii* and three comparator *Anopheles* species: *A. merus* (A), *A. christyi* (B), and *A. epiroticus* (C). *dN/dS* were assessed among tissue types (carcass and testes) based on expression profile (expressed, enriched, or specific). The width of each box-plot is proportional to the square root of the sample size. Regardless of the species used for comparison, testes-biased genes show elevated *dN/dS* values relative to genes with other expression profiles.

**Figure 4:**
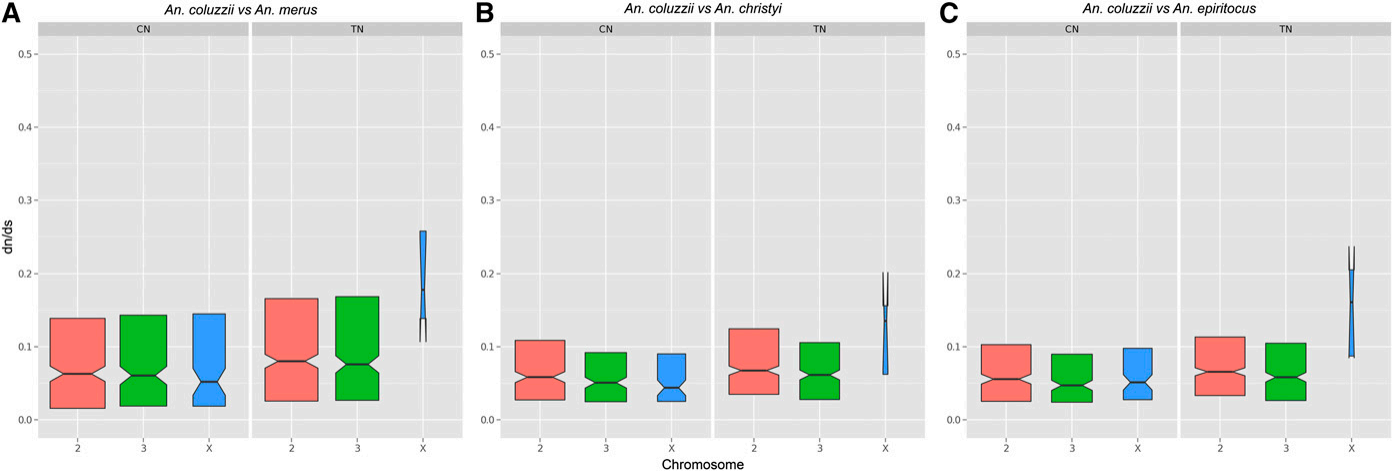
X-linked testes-biased genes exhibit extraordinary rates of evolution (A–C). Notched box-plots display the median, 25th, and 75th percentile values of *dN/dS* ratios (untransformed) between *A. coluzzii* and three comparator *Anopheles* species: *A. merus* (A), *A. christyi* (B), and *A. epiroticus* (C). *dN/dS* were assessed for tissue-enriched genes on each chromosome (TN and CN). The width of each box-plot is proportional to the square root of the sample size. TN genes located on the X show elevated *dN/dS* values relative to autosomal TN genes. Similar accelerated X-linked evolution is not seen in carcass-biased genes.

We next examined *dN/dS* in genes with different tissue profiles by comparing *A. coluzzii* to three different *Anopheles* species: *A. merus*, *A. christyi*, and *A. epiroticus*. The *dN/dS* were significantly affected by comparator species (*F*_2,34985_ = 6.639; *P* = 0.0013), tissue (*F*_1,34985_ = 45.423; *P* < 0.0001), expression profile (*F*_2,34985_ = 100.778; *P* < 0.0001), and a tissue-by-expression profile interaction (*F*_2,34985_ = 20.547; *P* < 0.0001). The *dN/dS* values for testes genes were consistently higher than for carcass genes, especially for TS genes, which were nearly twofold higher than CS genes (Figure 3). In other eukaryotes, it has previously been observed that X-linked male-biased genes show elevated *dN/dS* values relative to autosomal male-biased genes (Baines *et al.* 2008). To test for this pattern in *Anopheles*, we calculated *dN/dS* separately for TN genes on each chromosome (too few X-linked TS genes existed to conduct a robust analysis) (Figure 4). Indeed, we found that X-linked TN genes had roughly twofold higher *dN/dS* values than autosomal TN genes. X-linked CN and CS genes did not show elevated *dN/dS* values relative to their autosomal counterparts, proving that this pattern is specific to male-biased X-linked genes. The specific forces driving this rapid evolution of X-linked testes-biased genes compared to autosomal genes and X-linked carcass-biased genes are unclear, but might be due to the constant exposure of these genes to selection in heterogametic males.

#### Testes-biased genes are more likely to be differentially expressed:

We next determined if testes-biased genes (TN and TS) are more likely to be differentially expressed between *A. coluzzii* and *A. merus* than genes with other expression profiles. After filtering the gene lists to contain only *A. coluzzii* genes that have one-to-one *A. merus* orthologs, we calculated the number of differentially expressed genes (FDR < 0.05) between species for each tissue (*i.e.*, between *A. coluzzii* and *A. merus* testes and between *A. coluzzii* and *A. merus* carcass) (Table 3). The frequency of differentially expressed genes was analyzed using a generalized linear model with binomial error, with fixed effects of tissue type and expression profile. Results showed significant effects of tissue type (χ^2^ = 42.345; df = 1; *P* < 0.0001) and expression profile (χ^2^ = 24.969; df = 2; *P* < 0.0001) on the frequency of differentially expressed genes. Overall, 54% of TX genes are differentially expressed between *A. coluzzii* and *A. merus* testes. Both TN (63.1%) and TS (69.7%) genes were more likely to be differentially expressed than TX genes as a whole. In the carcass, only 45% of CX genes were differentially expressed between *A. gambiae* and *A. merus*. While carcass-biased genes (CN, 52%; CS, 50%) were more likely to be differentially expressed than CX genes, they were much less likely to be differentially expressed than testes-biased genes.

**Table 3:** The number of differentially expressed genes (DEGs) between *A. coluzzii* and *A. merus*, based on expression profile

	TX	TN	TS	CX	CN	CS
Total genes*^a^*	5006	2136	320	4663	1637	225
DEGs, *n* (%)	2703 (54)	1348 (63)	223 (70)	2111 (45)	845 (52)	113 (50)

a Total number of orthologous genes for a given expression profile.

#### High abundance of unannotated testes transcripts:

Due to the rapid evolution of testes-biased genes, we hypothesized that many testes transcripts are unannotated in the current genome assembly, which primarily relies on pairwise ortholog prediction from conserved sequence. Indeed, our GO enrichment analysis revealed the most significant enriched category of testes-specific *A. gambiae* genes was “unclassified.” To further test this hypothesis, we performed *de novo* assembly of the testes transcriptome and mapped the contigs back to the genome. In line with our expectations, we found that 2687 out of 15,330 total contigs mapped to regions of the genome not currently annotated as genes. Of these 2687 contigs, nearly half (49.6%) had open reading frames covering >50% of the assembled transcript, suggesting that many contain protein-coding genes. We can reasonably speculate that the rapid evolution of testes-biased genes and lack of orthologs is a major reason behind the poor annotation of these genes in the *A. gambiae* genome. Remarkably, many new genes across diverse taxa also show a male-biased expression, and the majority of these are specifically expressed in the testes (Dubruille *et al.* 2012). Many hypotheses have been proposed for this trend of new genes to be testes-specific, including male sex chromosomes inactivation and interaction between dosage compensation and sex-biased expression (Kaessmann 2010; Gallach and Betrán 2016).

### No evidence for additional Y genes

Recent studies have identified a variety of Y-linked sequence features in *A. gambiae* (*e.g.*, retrotransposon, satellite, ncRNA, and mRNA sequences) (Hall *et al.* 2013, 2016); however, the list is considered incomplete. We therefore searched for additional Y-linked sequences within our dataset using two approaches. The ABYSS *de novo* testes transcriptome assembly contained 31 contigs that completely failed to map to the reference genome and 377 contigs that mapped to scaffolds that have yet to be assigned to chromosomes (Table S4). We hypothesized that some of these genes may be located on the unassembled, heterochromatic Y chromosome. From this list, we tested the 32 contigs that had ≥10-fold higher expression in the testes than carcass for Y-linkage by PCR of individual male and female *A. coluzzii*. We found no evidence that any of the 32 transcripts are exclusively Y-linked; 16 amplified in both sexes, while 16 did not amplify in either sex.

In a second effort to identify new Y-linked genes, we generated a *de novo* transcriptome assembly via CLC bio using reads exclusively from testes libraries that either mapped to unplaced scaffolds or failed to map to the genome altogether. Assembly using this subset of reads resulted in 98 transcript contigs (Table S5), from which we identified five contigs that were testes-specific and another 14 contigs that had >eightfold higher expression in the testes relative to the carcass (Table S6). We tested all 19 of these testes-biased contigs for Y-linkage using PCR but none amplified exclusively in males.

Focusing on the eight previously identified Y-linked *A. gambiae* mRNA sequences (*YG1-8*) (Hall *et al.* 2013, 2016), two have been assigned a genome transcript ID (*YG7*: AGAP010291-RA; *YG8*: AGAP008501-RA). Our DESeq analysis revealed AGAP010291 is testes-specific, whereas AGAP008501 is expressed at similar levels in the testes and carcass, demonstrating that the expression of Y-linked genes is not necessarily limited to the testes. The remaining six Y-linked genes (*YG1-6*) were not found in our 98-contig subset, suggesting their expression levels were too low and/or our read mapping parameters were too stringent. However, both *YG1* and *YG5* mapped to contigs in our *de novo* ABYSS testes transcriptome. It is possible that additional Y-linked mRNA sequences are contained in this dataset but their identification will require further testing using lower stringency criterion.

### Conclusions

The testes have played a critical role in the solidification and preservation of species boundaries in the *A. gambiae* complex as hybrid male sterility is often the only postzygotic reproductive barrier between sibling species within the complex. In this study, we characterized and compared the testes and carcass transcriptomes of *A. coluzzii* and *A. merus*, two sibling species in the *A. gambiae* complex. As expected, we found that testes-biased genes are rapidly evolving at both the sequence and expression level, which is likely the cause of their relatively poor annotation in the reference genome assembly. Moreover, our results provide support that dosage compensation does not occur in the *Anopheles* male germline and, in fact, our results are consistent with chromosome-wide transcriptional repression of the X via an MSCI-like mechanism, as observed in other eukaryotes. Overall, our study is the first to fully characterize anopheline testes transcriptomes, and provides important insights into the linkage, function, and evolution of genes involved in *Anopheles* sperm and testes development. Importantly, this study has uncovered a long list of candidate male fertility genes, some of which may prove to be novel targets for malaria vector control.

## Supplementary Material

Supplemental material is available online at www.g3journal.org/lookup/suppl/doi:10.1534/g3.117.040089/-/DC1.
