# Supplementary material for: Comparative Transcriptomics of Malaria Mosquito Testes: Function, Evolution, and Linkage
Source: G3 (Bethesda). 2017 Feb 2;7(4):1127–36. doi: 10.1534/g3.117.040089 (PMC5386861; doi:10.1534/g3.117.040089)
Supplement: Supplementary file 1 [file 1127FigureS1.pptx]

## Slide 1
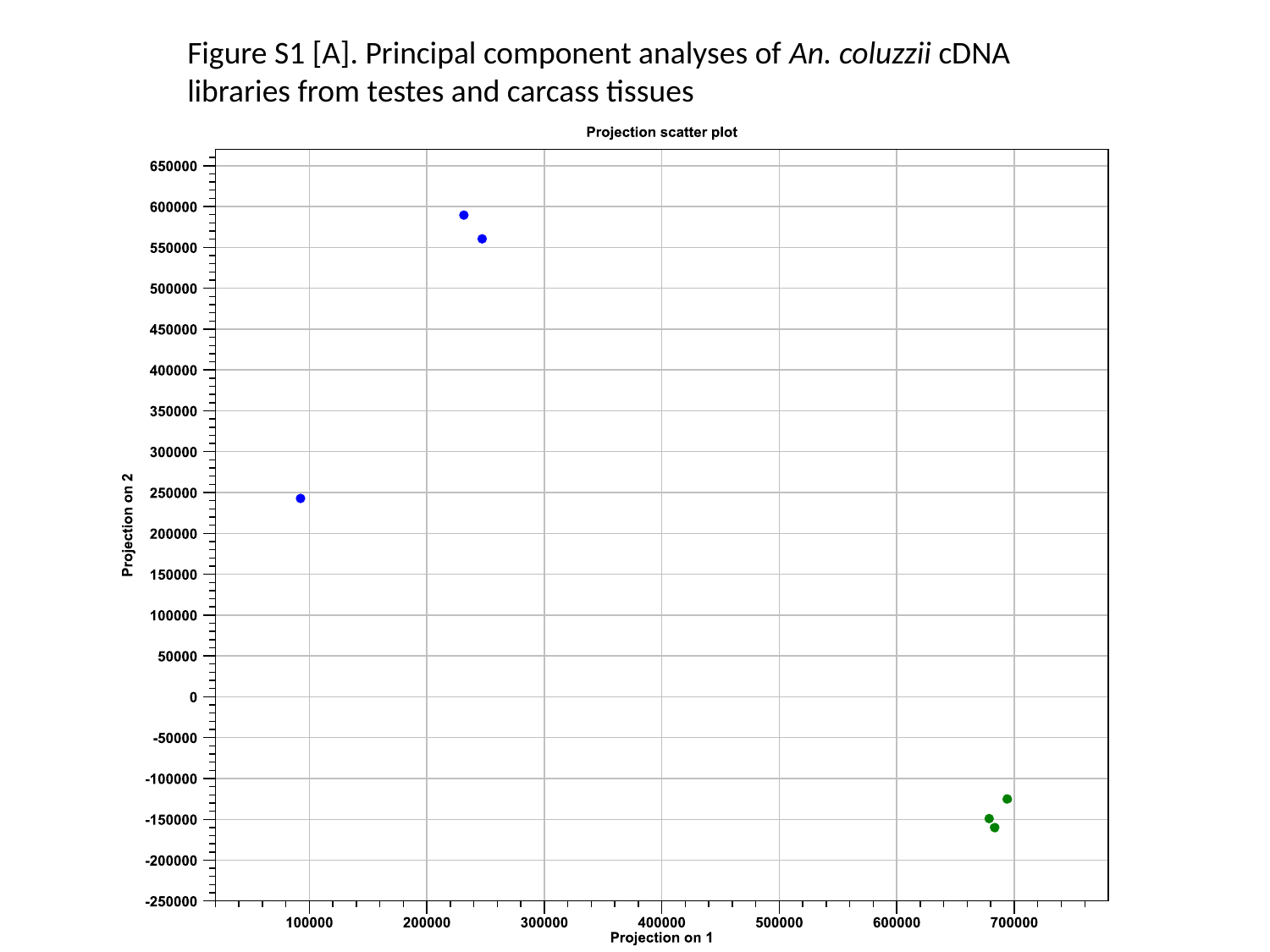

Figure S1 [A]. Principal component analyses of An. coluzzii cDNA libraries from testes and carcass tissues

## Slide 2
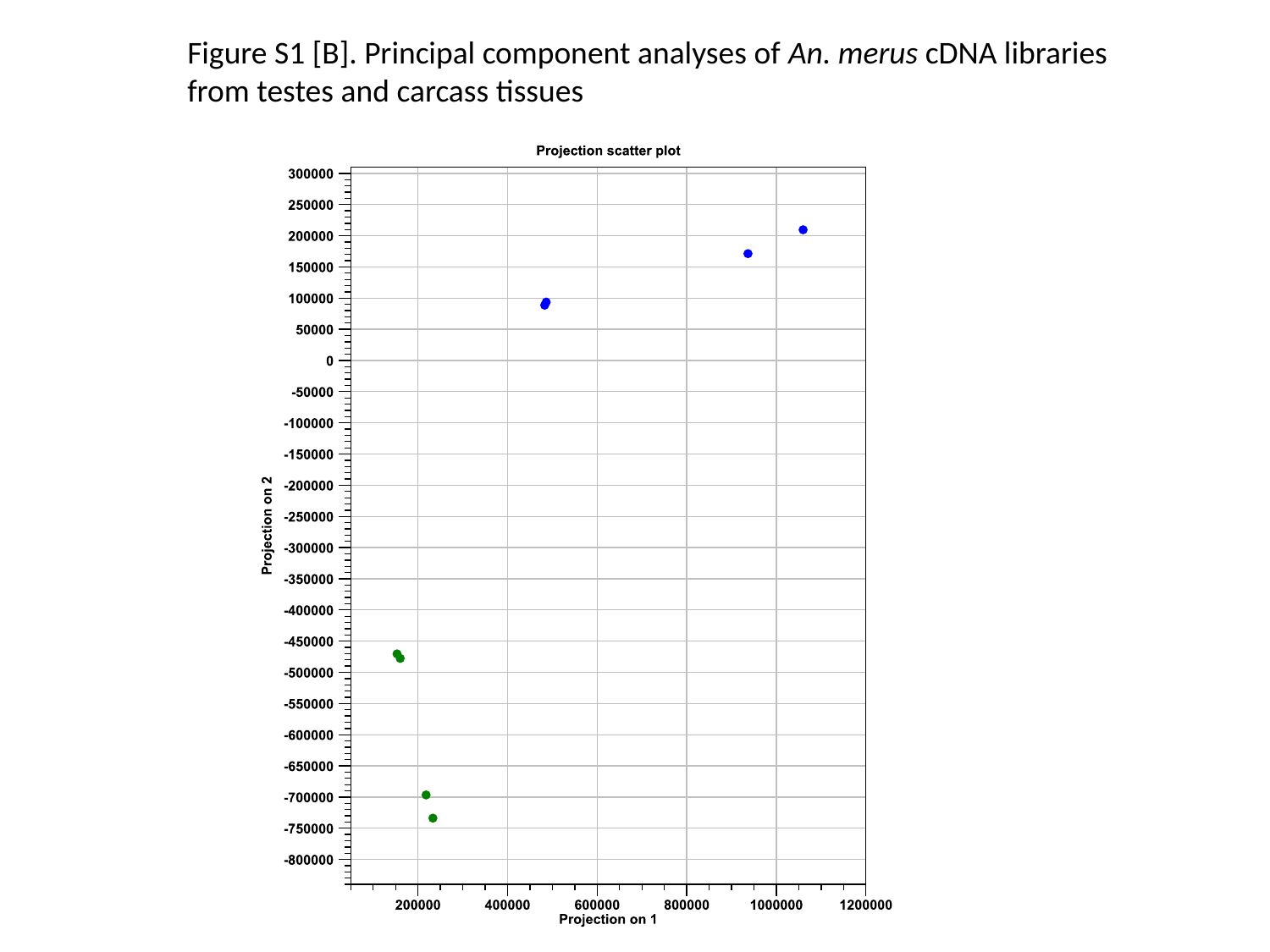

Figure S1 [B]. Principal component analyses of An. merus cDNA libraries from testes and carcass tissues
