# Supplementary material for: Comparative Transcriptomics of Malaria Mosquito Testes: Function, Evolution, and Linkage
Source: G3 (Bethesda). 2017 Feb 2;7(4):1127–36. doi: 10.1534/g3.117.040089 (PMC5386861; doi:10.1534/g3.117.040089)
Supplement: Supplementary file 2 [file 1127TableS1.docx]

Table S1 – Chromosomal distribution of genes significantly upregulated and downregualted in testes.

| Species | Chromosome 2 | | Chromosome 3 | | X chromosome | |
| --- | --- | --- | --- | --- | --- | --- |
|  | Up | Down | Up | Down | Up | Down |
| *An. coluzzii* | 478 | 1054 | 364 | 784 | 290 | 4 |
| *An. merus* | 535 | 974 | 376 | 685 | 220 | 6 |
