# Supplementary material for: Comparative Transcriptomics of Malaria Mosquito Testes: Function, Evolution, and Linkage
Source: G3 (Bethesda). 2017 Feb 2;7(4):1127–36. doi: 10.1534/g3.117.040089 (PMC5386861; doi:10.1534/g3.117.040089)
Supplement: Supplementary file 3 [file 1127TableS2.docx]

Tables S2. X–linked testes specific genes

| TS Gene(s) | Ovary | Paralogs | Drosophila Ortholog |
| --- | --- | --- | --- |
| AGAP000071 | No | AGAP000072 (X – TS*)  AGAP002488 | *CoRest*  FBgn0028343 |
| AGAP000133  AGAP000134  AGAP000135 | Yes | AGAP028171 (3R –TN) | N/A |
| AGAP000216 | No | 17 (16 AUTO; 1 UNK) | N/A |
| AGAP000421 | N/A | AGAP011534 (3L – TN) | *lethal (1) G0222* FBgn0028343 |
| AGAP000628  AGAP013360 | N/A | AGAP012340 (3L – TS) | *Qin*  FBgn0263974 |
| AGAP000817  AGAP012997  AGAP013424 | No | AGAP000816(X – TS*)  AGAP013173(X – TS*) | N/A |
| AGAP012998  AGAP013104  AGAP013235  AGAP013428  AGAP013444 | N/A | AGAP012536 (UNK – TN) | N/A |
| AGAP013399 | No | [AGAP000186](https://www.vectorbase.org/Anopheles_gambiae/Gene/Summary?db=core;g=AGAP000186) (X – TN)  [AGAP004515](https://www.vectorbase.org/Anopheles_gambiae/Gene/Summary?db=core;g=AGAP004515) (2R – TN) | N/A |

*Expression is only present in testes, but below cutoff that weestablished for expressed genes
